# The Role of Corticotropin-Releasing Hormone at Peripheral Nociceptors: Implications for Pain Modulation

**DOI:** 10.3390/biomedicines8120623

**Published:** 2020-12-17

**Authors:** Haiyan Zheng, Ji Yeon Lim, Jae Young Seong, Sun Wook Hwang

**Affiliations:** 1 Department of Biomedical Sciences, College of Medicine, Korea University, Seoul 02841, Korea; haiyan33@korea.ac.kr (H.Z.); ljyangel1004@korea.ac.kr (J.Y.L.); jyseong@korea.ac.kr (J.Y.S.); 2 Department of Physiology, College of Medicine, Korea University, Seoul 02841, Korea

**Keywords:** corticotropin-releasing hormone, corticotropin-releasing hormone receptor, nociceptor, dorsal root ganglion, pain

## Abstract

Peripheral nociceptors and their synaptic partners utilize neuropeptides for signal transmission. Such communication tunes the excitatory and inhibitory function of nociceptor-based circuits, eventually contributing to pain modulation. Corticotropin-releasing hormone (CRH) is the initiator hormone for the conventional hypothalamic-pituitary-adrenal axis, preparing our body for stress insults. Although knowledge of the expression and functional profiles of CRH and its receptors and the outcomes of their interactions has been actively accumulating for many brain regions, those for nociceptors are still under gradual investigation. Currently, based on the evidence of their expressions in nociceptors and their neighboring components, several hypotheses for possible pain modulations are emerging. Here we overview the historical attention to CRH and its receptors on the peripheral nociception and the recent increases in information regarding their roles in tuning pain signals. We also briefly contemplate the possibility that the stress-response paradigm can be locally intrapolated into intercellular communication that is driven by nociceptor neurons. Such endeavors may contribute to a more precise view of local peptidergic mechanisms of peripheral pain modulation.

## 1. Introduction

Pain and touch sensations occur through the relay of electrical impulses generated by somatosensory neural circuits, which comprise peripheral and central components [1]. In this circuit, peripheral nociceptors are among the primary afferent neurons that innervate local tissues and form synapses with the central components. Most of the nociceptors have their somas in the spinal dorsal root and trigeminal ganglia, and respond to potentially harmful physical or chemical changes in the innervated tissues, which then relay signals to the spinal cord or medulla.

Many molecular factors including neuropeptides tune nociceptor sensitivity in an autocrine or paracrine manner. Because pain exacerbation or attenuation often arises from such tuning, knowledge of their identities and tuning mechanisms at work may help not only to better understand the physiological aspect of pain, but also to devise pain control strategies that target nociceptors [2].

Local neuropeptides such as calcitonin-gene related peptide (CGRP) and a tachykinin substance P (SP) are currently well known to be secreted by nociceptors and act as slow neurotransmitters, which lead us to experience tonic pain whereas the typical ones like glutamate and aspartate evoke sharp and fast pain when released synaptically. Moreover, those local peptides may strengthen synapses in the spinal cord or medulla and may occasionally cause peripheral neurogenic inflammation when retrogradely secreted toward innervated tissues or blood vessels [3]. Gastrin-releasing peptide (GRP) is known to be an excitatory neuropeptide that is released from the pre- and post-synapses of proprioceptors, which are now considered as a separate population that relays itch signals [4]. On the other hand, major actions of some other neuropeptides including neuropeptide Y (NPY) and galanin (GAL) were shown to inhibit nociceptor excitability [5,6].

Information on the localized interactions of peptide hormones is also increasing. As well as CGRP and SP, nociceptors can also secrete pituitary adenylate cyclase-activating peptide (PACAP) and vasoactive intestinal peptide (VIP) to tissues when hyper-activated and aggravate neurogenic inflammation via vascular dilation and lymphocyte activation [7]. Bradykinin from damaged or inflamed tissues can directly fire nociceptors, resulting in pain exacerbation [8]. On the other hand, centrally generated opioid peptides such as enkephalins, endorphins, and dynorphins act as an inhibitory signal against nociceptor excitability on axo-axonic synapses in the spinal cord [9]. Such inhibitory actions in the spinal nociceptor synapses were also reported for arginine vasopressin (AVP), somatostatin (SST), and oxytocin (OXT) [10,11]. Recently, angiotensin II from inflamed tissues was shown to exert modulatory actions on nociceptor excitability although the outcomes seem to be different and dependent upon receptor subtypes expressed by nociceptors [12,13]. While these peptidergic actions are established to an extent in terms of their outcomes and receptor-mediated mechanisms in the nociceptor-based circuit, those of corticotropin-releasing hormone (CRH) are relatively primitive. Here, we overview the locations, roles, mechanisms, and hypotheses regarding how CRH and its receptors control pain transmission with up-to-date knowledge, focusing on the nociceptors.

## 2. Effects of CRH on Pain

CRH is one of the most upstream players in the hypothalamic-pituitary-adrenal (HPA) axis of hormonal networks. CRH, when released from the hypothalamus upon stress, evokes the subsequent secretion of adrenocorticotropic hormone (ACTH) by stimulating the anterior pituitary. ACTH circulates, reaches, and stimulates the adrenal gland, which finally secretes corticosteroids, preparing many different tissues and cells to cope with stress including pain [14]. Besides this main axis, secreted CRH also was shown to travel to the central nervous system (CNS) and affect its stress-associated function including pain modulation [15]. In addition, CRH outside the CNS also exhibited analgesic efficacy [16,17,18,19]. Research on CRH’s association with pain modulation has long undergone the confirmation of its analgesic effects, being occasionally accompanied by investigations on possible target regions within the context of the CNS versus non-CNS, for example, periaqueductal grey and immune cells, both of which are potentially related to analgesic opioid release [20,21]. The roles of nociceptors can be hypothesized for such potential CRH-mediated analgesic mechanisms regarding any of the CNS and non-CNS frame because of its transitional position, but this information is still emerging. Furthermore, it might also be interesting to investigate whether CRH acts in a similar fashion as the neuropeptides or hormones mentioned above affect nociceptor functions. Expressions and functions of CRH, its receptors, and endogenous ligands for the receptors in nociceptors may give insights into how CRH exerts its pain-modulatory action at cellular and molecular levels.

## 3. Expression of CRH in Nociceptors

Even earlier than maturation of the information on CRH and its receptors, a multitude of interesting approaches proposed identification of CRH-like molecules that possibly imply dorsal root ganglion (DRG) or nearby tissues as a location. When incubated with the duck spinal cord extract in vitro, the dissected pituitary generated ACTH, which suggests that components of the spinal cord, in which the central termini of nociceptors form synapses, contain some stimulant molecules that may or may not be CRH [22]. The medulla, the area that has synapses with trigeminal nociceptors, once failed to show CRH-like activity in a functional assay using rats [23]. But soon after the cloning of CRH [24], immunohistochemistry became active and a different group using CRH-targeting antibodies showed that CRH-like immunoreactivity in rat pons-medulla was quantitated about ~20% level, compared to that in the hypothalamus [25] (Table 1). An immunohistochemical approach targeting extended regions in the rat brain showed that some nerve termini but no cell body in the superficial laminae of the spinal cord and spinal nucleus of the trigeminal complex in the medulla displayed CRH-like immunoreactivities at many different levels [26]. This suggested that CRH is relatively abundant in presynapses of nociceptors rather than postsynapses of projection neurons or interneurons. This finding has been largely reproduced and some other expressions in dorsal horn neurons and descending fibers in the spinal cord were also reported in the next year [27,28]. Ablation of transient receptor potential vanilloid subtype 1 (TRPV1)-(+) neurons using neonatal capsaicin treatment confirmed nociceptor expression of CRH at the spinal and trigeminal levels in the rats [29].

Sensory ganglionic immunostaining eventually confirmed dorsal root and trigeminal ganglionic neurons, which appeared to have relatively smaller diameter somas, indicating that probably C- and Aδ-fiber nociceptors predominantly express CRH [35]. Immunocytochemistry using cultured or freshly dissociated DRG neurons also showed that 10–35% of neuronal populations were CRH-positive, and were mostly nociceptors because their soma sizes were small-to-medium diameter [34]. The De Groat lab that looked at feline spinal cords also readily detected moderate CRH-like immunoreactivity in somatosensory fibers located in the lumbar spinal dorsal horn as well as several numbers of superficial nuclei in it, reproducing the results obtained from rat studies so far [30]. Korosi et al. also repeated CRH expressions in fibers that terminated in the superficial laminae as well as the deeper ones when they immunostained the murine spinal cord [32].

To date, two pathologic models have been tried for CRH expression in nociceptors. The Hwang lab, before they used spinal nerve injury (SNI)-induced neuropathic pain models, confirmed by using immunohistochemistry that a number of small-diameter DRG neurons that were presumably among nociceptors were CRH-positive under normal condition [31]. These CRH-(+) DRG neurons were also confirmed to express TRPV1. Interestingly, under SNI-neuropathic conditions, animals exhibited some changes in expression including robust increases in CRH and TRPV1 expression in contralateral small-diameter DRG neurons and decreases in ipsilateral neurons. These differences again occurred in fiber staining in the corresponding spinal laminae: the contralateral side showed greater expression than the ipsilateral one. These results suggest that changes in CRH expression may be associated with SNI-neuropathic pain in some way. The reduction in the number of nociceptors owing to neuropathic degeneration itself may explain the ipsilateral decrease in CRH expression as observed in other previous studies [47,48,49].

Later, an approach to sorting out potentially pain-associated genes has generated interesting data. Quantitative analyses on mRNA levels in DRG neurons from chronic constriction injury (CCI)-induced neuropathic pain models using mice found dozens of genes with altered expression, particularly in neuropathic neurons compared to undamaged contralateral or adjacent neurons, and the most increased one was *crh* [33]. Thus collectively, studies inquiring the presence of CRH, throughout the history of DRG research commonly argued that at least a subpopulation of nociceptors contain CRH, which may potentially be important in peripheral pain processing by nociceptor-involved circuit.

This concept may require an integrated view regarding receptor expression, and we overview this in the next section.

## 4. Expression of CRH Receptors in Nociceptors and Their Neighbors

The CRH receptors (CRHRs) are among members of the secretin-like (class B) family of G-protein-coupled receptors (GPRs) and there are two subtypes known as CRHR1 and CRHR2 [14]. Even before these were cloned in the mid-1990s [50,51,52,53,54,55], the receptors were predicted to be among surface receptors and anatomical and functional studies began as soon as the localization of CRH in nociceptors was repeated. Two independent autoradiographic approaches using a ^125^I-labeled ovine CRH were the first to show its specific binding in the superficial dorsal horn of the spinal cord and the central termini of nociceptors in rats [35,38] (Table 1). De Souza et al., also observed binding in the spinal tract of the trigeminal nerve and its nuclei that appeared to show relatively robust bindings compared to that in the spinal cord. A subsequent combined approach using autoradiography and electrophysiological recording of the ventral roots showed that putative receptor activation in the dorsal horn by cord perfusion with CRH elicited circuit-dependent depolarization of the motor components [37].

After a series of cloning reports in 1993 and 1995, the Vale lab first reported the spinal trigeminal nucleus as a major expression site for CRHR1, which was confirmed by Rivest et al., suggesting a possible interaction with a ligand secreted from neighbor regions and the potential nociception-related role of this receptor [40,41]. CRHR2 expression was later assessed. When a radiolabeled CRHR2-specific ligand ^125^I-K41498 was used, CRHR2 expressions in the rat spinal trigeminal nucleus as well as in the nucleus tractus solitarius and area postrema that seem to play a primary sensory role despite not being a cognitive one were readily detected [45]. mRNA detection for *crhr2* was also successful in a reverse transcriptase-polymerase chain reaction (RT-PCR) analysis using rat lumbar DRGs as well as the relevant spinal cord section [44].

Korosi et al. aimed to show the laminae distribution of *crhr1* and *crhr2* in their in situ hybridization assays using murine spinal cords [32]. Interestingly, although those two subtypes both exhibited wide distribution throughout the laminae, those were faint in the superficial laminae area, in which CRH-(+) nociceptors mainly terminate their axons. Controversial results were again obtained from immunohistochemistry. The Hwang group not only traced CRH expression as mentioned above, but also CRHR distribution in the rat DRG and spinal cord [31]. CRHR1 was not detectable in DRGs and the dorsal horn in normal naïve rats but under an SNI-neuropathy model, it was seen in the activated microglia of the deep laminae. Although less informative in the spinal cord, CRHR2 expression was weak in the DRG of the naïve animals and became apparent only in the contralateral DRG under SNI conditions. Regional discrepancies between ligand-containing termini and receptor-containing soma may raise other possibilities for ligand-receptor partnering. For example, urocortins (UCNs) and a frog version sauvagine are also endogenous neuropeptides that can activate CRHRs and their relatively broad expressions throughout the laminae have been reported in the spinal cord [36,56]. However, further confirmation is required as to whether those different ligands, probably originating not only from nociceptors but also innocuous collaterals of mechanoreceptors located in deeper laminae, are major interactors with CRHRs and how much those interactions, if any, affect pain transmission. Based on such increasing evidence for CRHR, functional approaches that investigate the effect on peripheral nociception have increased as mentioned below.

## 5. Outcomes from CRHR Activation in the Peripheral Circuit

Even in the early stage when most studies covered expressions, Wei et al. focused on a functional aspect. When they observed the rat paw, CRH administration inhibited neurogenic extravasation, which is one of the important mechanisms of nociceptor-induced neurogenic inflammation [57]. This inhibitory effect did not appear to depend on the HPA-axis or opioids because it was not blunted by adrenalectomy, hypophysectomy, or naloxone treatment. This HPA-axis and opioid independent effects were also seen in their extracellular recording of wide dynamic range (WDR) neurons in the rat spinal trigeminal nucleus, in addition to nociceptive sensitivity to CRH [43] (Table 1). Interestingly, peripheral heat-evoked discharges were significantly decreased while spontaneous discharges of cold-specific WDR neurons were increased by CRH and treatment with α-helical CRF 9-41 that is now known as a non-specific CRHR antagonist reversed both of these effects, indicating that CRH was able to drive its receptor-mediated but modality-specific differential outcomes. Condition-specific differential effects were detected later. When Ikeda et al. recorded lamina II neurons in rat spinal cord slices, CRH perfusion facilitated A-fiber-mediated neuronal responses upon high frequency stimulation but suppressed it under a GABA-incapacitated condition [42]. These data possibly suggest that CRH may display diverse effects depending on cellular subpopulations that selectively express a receptor isotype, if any, although blunted nociception was commonly observed.

**Figure 1:**
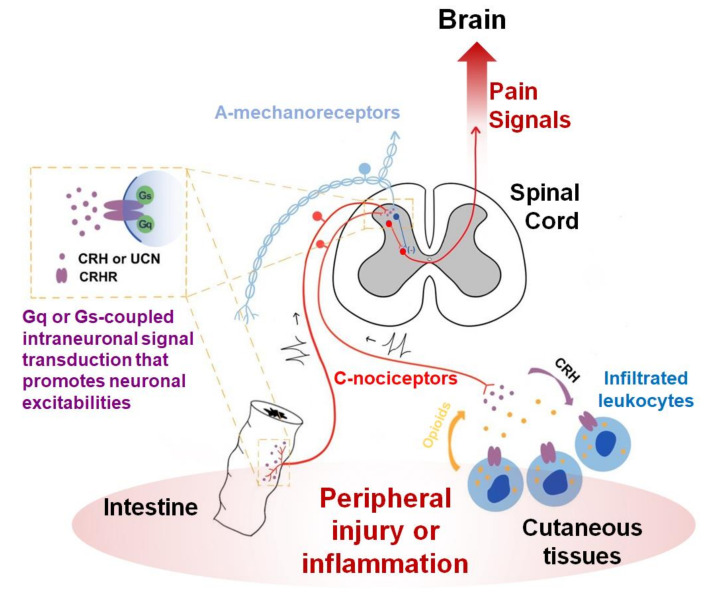
A conceptual diagram depicting the role of CRH and its receptors in the peripheral nociceptor-based circuit. Injured or inflamed intestines may locally secrete CRH, stimulating CRHRs in the peripheral termini of nociceptors, which can promote their excitations via Gs- or Gq-protein α subunits-mediated intracellular signaling. By contrast, CRH antidromically released from those termini can occasionally activate CRHRs in infiltrated immune cells, dampening nociceptor signals by providing endogenous opioids. CRH released from the central termini of nociceptors may possibly activate interneuronal CRHRs in the superficial laminae in the spinal cord and then pain transmission can be attenuated. Spinal deep laminae are also known to express CRHRs but the cellular components that stimulate those by secreting ligands remains to be determined.

Mousa et al. systematically compared the CNS and peripheral effects of CRH injection on pain using three different local routes (intracerebroventricular, intrathecal, and intraplantarly routes) in one study and all of those exhibited analgesia at doses that unlikely diffuse to other regions at a significant level [21]. The effectiveness of intrathecal and intraplantarly injection implies that the local interaction between CRHRs and their ligands may endogenously occur near the nociceptors. In fact, the same group has worked on local mechanisms in the periphery. They hypothesized that some subtypes of leukocytes recruited under inflammation or nerve injuries can secrete opioids in response to increased CRH possibly produced from nociceptors or their neighbors and that those peripheral opioids may reduce nociceptor excitability [58,59] (Figure 1). CRHRs were detected in leukocytes using ligand binding and immunostaining assays [60,61]. Endorphins and endomorphins appear to be among the major opioids released from infiltrated immune cells upon CRHR activation [62,63,64]. While the possibility remains open as to whether the ligands originate from nociceptor neurons in their studies, the receptors were only seen in the immune components but not in the nociceptor neurons. With respect to this leukocyte-involved mechanism, receptor subtype information for the CRHR still remains unknown. A study at a morphological aspect showed that CRH’s effect on DRG neurons was again indirect. DRG neuronal outgrowth was enhanced, which is likely explained by microglial brain-derived neurotrophic factor (BDNF) release upon CRH stimulation [65]. But the CRHR subtype related to this effect also remains undetermined.

In the spinal cord, more or less controversial results have been reported. Intrathecal treatment with CRH and UCN2 effectively attenuated duodenal distension-induced pain behaviors and related visceromotor responses (VMRs) [46]. The CRH-induced analgesia was only reversed by antisauvagine-30, a relatively selective CRHR2 antagonist, but not by CP-154,526, a CRHR1-specific antagonist, indicating that spinal CRHR2 may be a major contributor to the analgesic effect. Similar results were independently obtained from a colorectal distension model using UCN2 [44]. On the other hand, when measuring the dorsal-root to ventral-root reflex of ex vivo lumbosacral spinal slices from rats, Aδ- and C-fiber nociceptor-mediated reflexes were dampened by CRHR1 activation [39]. It remains unclear whether those CRHR1 actions were exerted in nociceptors or at postsynapses. Although it was assumed that the source of the endogenous ligand might be cerebrospinal fluid, Larson et al. reported another contradictory result from the spinal cord. Intrathecal UCN injection elicited tactile allodynia in rats, which could not be reproduced by CRH injection, indicating that there is the possibility that an unknown and different binding partner for UCN might underlie this mechanism [66]. Nonetheless, CRHR1 antagonism was effective in blunting this pain status but that of CRHR2 was not. Collectively, phenotypical outcomes so far likely support this contribution to pain relief, but its interpretation seems to be still preliminary, and awaits thoroughly mechanistic explanations using more sophisticated approaches with refined hypotheses (Figure 1).

## 6. Unsolved Questions Regarding Peripheral Pain Transmission

Expression profiles predicted possible pain modulation by CRH and its receptors in the nociceptor-based circuit. However, many questions remain to be clarified, for example, how CRHR expressing cells undergo nociceptive insults, what the endogenous ligands result in, and how quantitatively and qualitatively these interactions contribute to pain modulation.

Largely, CRH has been detected in nociceptors while CRHRs mostly appear to be present in synaptic interactor neighbors such as dorsal horn neurons or innervated peripheral tissues. But at least a subpopulation of nociceptor neurons, particularly under pathological states including neuropathic conditions also seem to express CRHRs. This suggests that this ligand may play a role in an autocrine and/or paracrine manner, otherwise that the sensory neurons themselves may also have the potential to respond to CRH from other sources. Because both CRHRs are well known to drive Gs or Gq/11 signal transduction that typically promotes neuronal excitation, it can be assumed that those CRHR-(+) neurons, when stimulated by the ligands, may increase their firing and eventually contribute to pain aggravation, which has not been experimentally confirmed yet. In addition, CRHR2-specific Gi/o-coupled downstream first found in trophoblasts, may complicate its data translation [67]. More precise approaches that discriminate DRG neurons from other partner cells, seems to be required.

Regional discrepancies, as mentioned above, between ligand producer and receiver components in the spinal dorsal horn layers are another question. When exclusively focusing on superficial laminae, one can simply hypothesize that inhibitory interneurons, excited upon CRH from nociceptors, can downregulate nociceptive signals. Given this notion, it is unlikely to fully explain the raison d’être of broadly expressed CRHRs throughout the laminae. Possibly, complex local interactions between interneuronal ligands and receptors may exist. Otherwise, Aδ nociceptors, which form synapses also in the deeper laminae may contribute to CRH signals, particularly when their ligand expression is pathologically upregulated. Also, UCN positivity needs to be compared among whole DRG populations including mechanoreceptors as well as nociceptors because the possibility as to whether the collateral terminals of mechanoreceptors participate in the transmission based on the gate-control theory should be checked.

A comparative validation as an analgesic target is another way to interrogate neuropeptides and their receptors. Neuropeptides in nociceptors such as CGRP, PACAP, SP, VIP, GAL, NPY, VIP, SST, AVP, and OXT all appear to play a crucial role in pain transmission. On the other hand, their specificities to some pain phenotypes, the extent of their expression and also its breadth, and the quantities of their effects on pain intensity especially under pathologic conditions, frequently open or limit opportunities to employ proof of concept regarding their roles. Quantitative approaches for nociceptive synaptic transmissions and pain outcomes due to controlling CRH paradigms may help precise definition of its validity as an analgesic target. Accessibility to peptide and receptor expressers can also be important. Basically, nociceptors are surrounded by thinner blood brain barriers and even their peripheral termini are exposed to tissues, which can be an advantage in that regard [68]. Future understanding of which among the two nociceptor termini endogenously secrete CRH and if both, which among the subsequent spinal modulation and opioidergic control of infiltrated leukocytes is more potent, can be helpful to further assess target utility, considering the recent translational progress in biologic controls of CGRP and its receptor [69].

## 7. Perspectives from a Stress Viewpoint?

Besides the conventional HPA axis, many other tissues are now known to express CRH and its receptors, which appear to play a specific role dependent on location. Regarding pain transmission, not only the nociceptor-based circuit, which is reviewed here, but also other regions such as the amygdala in the CNS and the gastrointestinal tract in the innervated periphery draw attention as places where the ligand and receptor interact [70,71]. Despite different locations, circuits, and outcomes, a hypothesis can be made that the HPA concept may operate in a functionally similar fashion at a relatively local level. That is, CRH may be utilized as an alert messenger reporting stress given to its donor cells and the cells that express CRHRs may behave as effectors to resolve the problem as soon as they are stimulated by CRH. The effectors in the HPA axis generate ACTH and then cortisol, which leads our body to begin systemic preparation against stress. If nociceptors are hypothesized to be the alarm generators producing CRH, outcomes from the effectors, which those neurons have contact with, may be diverse and heterogenous, depending on what kind of actions the effectors exert when their CRHR is activated. This may indicate that the solutions finally offered by the effectors are also differential, based on pain mediation itself and the pain-producing damages the effectors have been evolved to recognize as a major stress problem that must be resolved in the first place.

Opioids-producing leukocytes may be an effector that dampens the pain mediation itself. So may spinal inhibitory interneurons although this has not been thoroughly confirmed. Regarding outcomes from the CRHR-mediated responses in visceral and vascular epithelia, also assumed to be effectors, their preparation might lean more on the removal of potential damage, because these generally promote inflammation or motility for the defense. This latter paradigm may help pain resolution from a relatively long-term perspective but can acutely aggravate pain owing to pro-inflammatory processes and hypersensitivity, particularly during the developmental stage of inflammation. This can be a possible explanation for why CRH antagonism, but not its agonism has been shown to contribute to pain reduction via epithelial CRHR activation in visceral hypersensitivity, endometriosis, and cystitis, etc. [71,72,73] (Figure 1). In other words, the removal of damage might be prioritized compared with pain reduction in those cases. This is still a hypothesis without evidence for the role of endogenous ligands during local interactions and, if any, even that of CRH that may distantly diffuse from the HPA axis. Functional interactions between the endogenous ligand-receptor expresser network in nociceptor-innervated local tissues needs to be confirmed first.

## 8. Conclusions

Although study of CRH has started at an early stage, the knowledge of the CRH function in peripheral nociception is immature compared to that for other neuropeptides. Not only the systemic effect of CRH from the HPA axis, its local and specific action occurring in the nociceptor-based circuit appears to be potentially important in pain modulation according to the accumulating evidence to date. It will be interesting to see whether the HPA paradigm of coping with stress can also be applied to pain transmission or neurogenic management of pain-producing damage. Future efforts defining CRH- and UCN-specific labeled lines, which likely arise more conspicuously under neuropathic or inflammatory conditions, may shed light on unknown aspects of the physiology of pathologic pain. This will not only academically contribute to the mapping of peripheral somatosensory circuits where other peptidergic information is currently preceding, but also therapeutically to the devising of new strategies for controlling pain diseases possibly by providing a novel proof of concept.

## Figures and Tables

**Table 1:** Expressions of corticotropin-releasing hormone (CRH) and CRH receptors (CRHRs) in dorsal root ganglia (DRG) and related components mentioned in this review. Abbreviations: UCN, urocortins; RT-PCR, reverse transcriptase-polymerase chain reaction

Molecules	Locations	Observations	References
CRH	DRG termini or spinal neurons	Functional analysis	[22]
	DRG termini	Immunostaining	[26,27,28,29,30,31,32]
	DRG ganglia	Immunostaining	[31,33,34,35]
		Quantitative PCR	[33]
	Trigeminal termini	Immunostaining	[25,26,29]
	Trigeminal ganglia	Immunostaining	[35]
	Spinal neurons	Immunostaining	[27,28,30]
UCN	DRG termini	Immunostaining	[36]
CRHRs	DRG termini	Ligand-labeling	[35,37,38]
(nonselective)		Functional analysis	[21,37]
	Trigeminal termini	Ligand-labeling	[38]
	Trigeminal nucleus	Ligand-labeling	[38]
CRHR1	DRG termini or spinal neurons	Functional analysis	[39]
	Trigeminal nucleus	In situ hybridization	[40,41]
	Spinal neurons	Functional analysis	[42,43]
	Spinal microglia	Immunostaining	[31]
CRHR2	DRG ganglia	RT-PCR	[44]
		Immunostaining	[31]
	Trigeminal nucleus	Ligand-labeling	[45]
	Spinal neurons	In situ hybridization	[32]
		Functional analysis	[46]
		Functional analysis	[44]
		RT-PCR	[44]
